# Relative Recovery of Non-Alcoholic Fatty Liver Disease (NAFLD) in Diet-Induced Obese Rats

**DOI:** 10.3390/nu16010115

**Published:** 2023-12-28

**Authors:** Hamda M. Aboujassoum, Vidya Mohamed-Ali, David Abraham, Lucie H. Clapp, Hamda A. Al-Naemi

**Affiliations:** 1 Laboratory Animal Research Center, Qatar University, Doha P.O. Box 2713, Qatar; halnaemi@qu.edu.qa; 2 Anti-Doping Laboratory Qatar, Sports City Road, Doha P.O. Box 2713, Qatar; v.mohamed-ali@ucl.ac.uk; 3 Centre of Metabolism and Inflammation, Division of Medicine, Royal Free Campus, University College London, Rowland Hill Street, London NW3 2PF, UK; 4 Centre of Rheumatology and Connective Tissue Disorders, Division of Medicine, Royal Free Campus, University College London, Rowland Hill Street, London NW3 2PF, UK; david.abraham@ucl.ac.uk; 5 Institute of Cardiovascular Science, University College London, Rayne Building, 5 University Street, London WC1E 6JF, UK; l.clapp@ucl.ac.uk; 6 Department of Biological and Environmental Sciences, Qatar University, Doha P.O. Box 2713, Qatar

**Keywords:** cafeteria diet, obesity, metabolic syndrome, non-alcoholic fatty liver diseases, hepatic steatosis

## Abstract

Consumption of a high-carbohydrate diet has a critical role in the induction of weight gain and obesity-related pathologies. This study tested the hypothesis that a carbohydrate-rich diet induces weight gain, ectopic fat deposition, associated metabolic risks and development of non-alcoholic fatty liver disease (NAFLD), which are partially reversible following carbohydrate reduction. Sprague Dawley (SD) rats were fed a carbohydrate-enriched cafeteria diet (CAF) or normal chow (NC) ad libitum for 16–18 weeks. In the reversible group (REV), the CAF was replaced with NC for a further 3 weeks (18–21 weeks). Animals fed the CAF diet showed significantly increased body weight compared to those fed NC, accompanied by abnormal changes in their systemic insulin and triglycerides, elevation of hepatic triglyceride and hepatic steatosis. In the REV group, when the CAF diet was stopped, a modest, non-significant weight loss was associated with improvement in systemic insulin and appearance of the liver, with lower gross fatty deposits and hepatic triglyceride. In conclusion, a carbohydrate-enriched diet led to many features of metabolic syndrome, including hyperinsulinemia, while a dietary reduction in this macronutrient, even for a short period, was able to restore normoinsulinemia, and reversed some of the obesity-related hepatic abnormalities, without significant weight loss.

## 1. Introduction

Diet is an important and modifiable risk factor for obesity and associated non-communicable diseases. Clinical recommendations have focused on the consumption of diets low in fat, with alternatives for high-fat components being widely marketed for the reduction in body weight and cardiovascular risk factors. However, since the 1970s, evidence showed that high-carbohydrate diets, particularly those high in simple sugars, facilitate weight gain, hyperinsulinemia and elevated risk of obesity-associated diseases [1]. Rats fed a high-carbohydrate diet showed progressive increases in body weight, abdominal fat deposition and impaired metabolic parameters [2,3]. In human studies, high-carbohydrate diets (50–70% energy intake) were associated with higher mortality compared to a high-fat diet [4].

Non-alcoholic fatty liver disease (NAFLD) is widely attributed to the obesity epidemic. It is defined as the accumulation of excessive fat in the liver, with the presence of >5% steatosis in individuals with no history of alcohol abuse [5]. The global prevalence of NAFLD is 29.8% [6]. Approximately 10–25% of patients who have a fatty liver end up with non-alcoholic steatohepatitis (NASH), and 5–8% of those patients might progress to liver cirrhosis in 5 years [7]. Excess dietary free fatty acids (FFAs) and visceral fat (VAT) appear the main contributors to NAFLD development. VAT is a key source of FFAs, mobilized through lipolysis and is directly deposited in the liver, inducing hepatic triglyceride synthesis, leading to hepatic steatosis [8].

Interestingly, dietary weight loss is one of the effective measures to reverse obesity-related abnormalities. A recent meta-analysis showed that a low-carbohydrate diet triggers weight loss more effectively than a low-fat diet [9]. Further, lifestyle modification markedly improved histological features of NAFLD [10], suggesting that weight loss might be the best treatment for NAFLD and NASH [11].

Thus, the aims of the present study tested the hypothesis that a carbohydrate-rich diet (CAF) induces weight gain, ectopic fat deposition, associated metabolic risks and the development of non-alcoholic fatty liver disease (NAFLD), which were partially reversible by CAF elimination.

## 2. Materials and Methods

### 2.1. Animals

**Figure 1:**
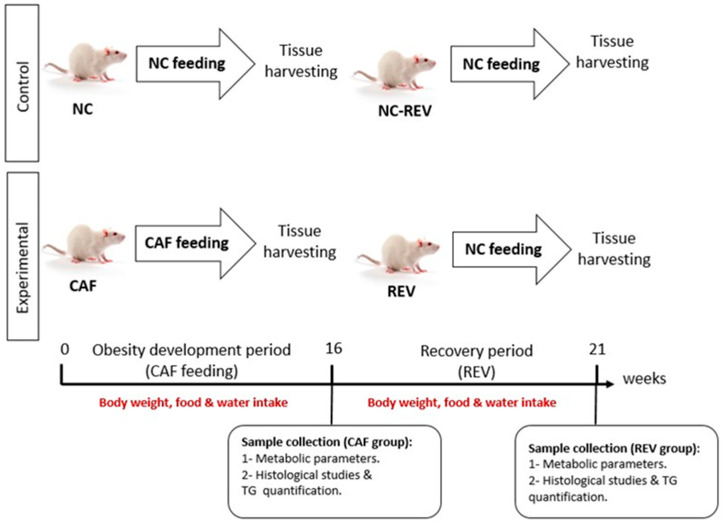
Study design. Figure illustrates the CAF feeding period, reversibility period and the time points of sample collection throughout the study. NC: represent the control group (fed with normal diet), CAF: represent the cafeteria diet group (fed with high carbohydrate diet), NC-REV: represent the control of reversible group (fed with Normal diet), and REV: Represent the REV group (fed with CAF diet, and then reverted to NC).

All experimental procedures were approved by the Institutional Animal Care and Use Committee at Qatar University (QU-IACUC# 019/2014) and performed at Qatar University Laboratory Animal Research Center (LARC), in accordance with the guidelines and regulations of the Guide for the care and use of Laboratory Animals published by the US National Institutes of Health. Seventy-nine male Sprague Dawley rats (Charles River, UK) were housed under controlled environmental conditions: temperature (20–23 °C ± 1 °C), humidity (30–70% ± 5%), and light/dark cycle (12:12). At 8–9 weeks of age, rats were weighed and randomly assigned to 3 animal cages for the following main groups: the NC diet group (control, n = 26), the cafeteria diet group (CAF, n = 26) and the reversible group (REV, n = 27), in whom the CAF diet was reverted to NC for 3 weeks after 18 weeks of CAF feeding. The NC group had ad libitum access to the NC diet (carbohydrate 49%, fat 4.6%, protein 14.4%), and regular water for 16–18 weeks. After this period, the NC group was further divided into another control group called NC-REV and used as a control for the REV group (Figure 1). The CAF group had ad libitum access to both the NC and CAF (carbohydrate 60–70%, fat 10%, protein 9–12%) diets, and water with 5% added sugar (sucrose) to represent soda drinks for the same time period (the total carbohydrate content represented the food and the sugary water). The CAF diet was provided freshly every day. Animals in the CAF group could select and consume freely either of the NC and CAF diets, which were provided in excess (see Supplementary Material: S1; Table S1). The REV group was treated the same as the CAF group but was switched at week 18 to NC and regular water for 3 weeks (weeks 19–21). Food and water intake as well as body weight were measured and recorded weekly throughout this study.

### 2.2. Tissue Dissection and Sample Collection

Animals were fasted for 12 h prior to dissection. Animals were anesthetized with 20 mg/kg Sodium pentobarbital (Ilium Thiopentone, Troy Laboratories Pty Ltd., Glendenning, Australia) intraperitoneally and blood was collected by cardiac puncture. Liver tissue samples were collected and washed with phosphate-buffered saline (PBS) before processing. Liver weight was recorded.

### 2.3. Metabolic Parameters

Serum samples were used to measure glucose levels, lipid profile (cholesterol, triglycerides, high-density lipoprotein cholesterol (HDL) and low-density lipoprotein cholesterol (LDL)) and markers of liver function (serum alanine transaminase, ALT; aspartate transaminase; AST; alkaline phosphatase, ALP) and albumin using a clinical chemistry analyzer (COBAS INTEGRA 400 plus, Roche Diagnosis, Rotkreuz, Switzerland). Insulin concentration was measured using a rat insulin enzyme-linked immunosorbent assay (ELISA) kit (Cat.#80-INSRT-E01, Alpco Immunoassays, Salem, NH, USA) as per the manufacturer’s instruction.

### 2.4. Hepatology

Liver samples were fixed in 4% buffered formalin for 24–48 h, washed with tap water, dehydrated in a serial dilution of ethanol, cleared with xylene, and embedded in paraffin wax. Paraffin wax blocks were sectioned at 4–5 μm thickness, placed on glass slides, deparaffinized and stained with two different stains hematoxylin/eosin, and Sirius red and examined by light microscopy. NAFLD scoring was evaluated blindly, based on three histological features: hepatocyte ballooning, micro- and macrovesicular steatosis and lobular inflammation, as described previously [12]. Five random fields from each section were evaluated as the following: hepatocyte ballooning (grade 0 = absent, grade 1 = mild, grade 2 = moderate, grade 3 = severe) hepatic micro- and macrovesicular steatosis (grade 0 ≤ 5%, grade 1 = 5–33%, grade 2 = 33–66% and grade 3 > 60%), lobular inflammation (grade 0 = absent, 1 = minimal, 2 = mild, 3 = moderate, 4 = severe). A colorimetric/fluorometric triglyceride quantification assay (Cat. #ab65336, Abcam) was used to measure hepatic triglyceride content according to the manufacturer’s instruction. In this assay, triglyceride is converted to free fatty acids and glycerol, then glycerol is oxidized and generates a product that reacts with the triglyceride probe to form a color at 570 nm. The detection range of the triglyceride assay was between 2 μM and 10 mM.

### 2.5. Statistical Analysis

Statistical analysis of the data was carried out using Prism^®^ software (GraphPad Prism version 8 for Mac OS X, Boston, MA, USA). Comparisons were between either NC vs. CAF or NC-REV vs. REV. Body weight, food and water intake were analyzed by two-way analysis of variance (ANOVA), followed by a repeated-measures protocol. The biochemical parameters were analyzed using Student’s *t*-test. All data were shown as the mean ± standard error of mean (SEM). Significance was defined as *p* ≤ 0.05.

## 3. Results

### 3.1. Body Weight

**Figure 2:**
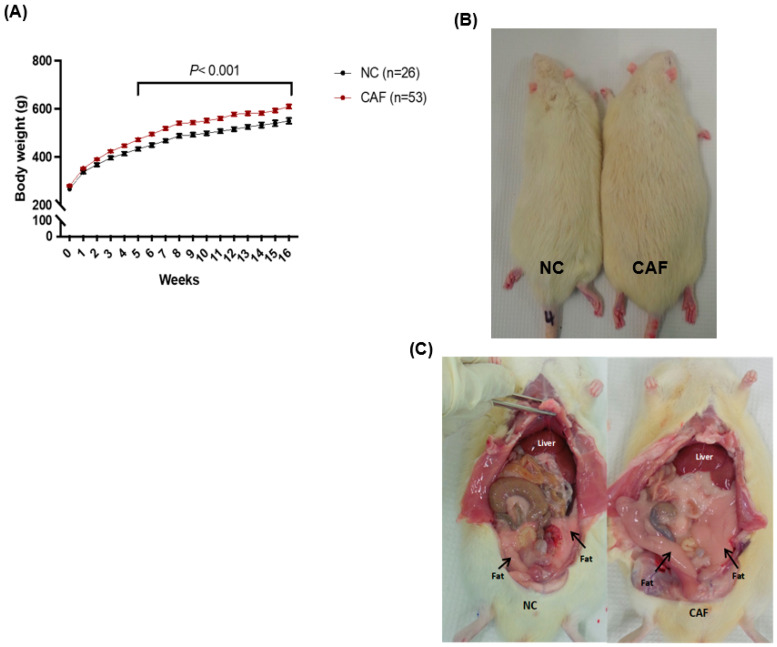
Effect of the CAF diet on body weight. Rats were fed daily for 16 weeks on NC (for NC group) and on both CAF and NC (for the CAF group). (**A**) The graph represents the average body weight of both groups NC and CAF. The CAF group showed a significant increase in body weight (red curve) compared to the NC group (black curve). (**B**) External body size of representative animals from the NC and CAF groups. (**C**) CAF-fed rats showed clear abdominal fat deposition in the abdominal cavity compared to NC-fed rats. Black arrows are pointing at fatty tissues in the abdominal cavity. Data were analyzed by two-way ANOVA with repeated-measures and expressed as the mean ± SEM. *p* < 0.001 denotes the statistical differences between NC and CAF.

Over a period of 16–18 weeks, CAF-fed rats gained significantly more weight compared to NC-fed rats (*p* < 0.001; Figure 2A). NC rats gained 290 g on average over 16 weeks, while CAF rats gained 350 g on average during the identical period. There is no threshold that has been established to diagnose obesity in animal models, such as those established for human. Thus, in this study, an animal that gained 20–30% weight compared to control was considered an obese animal, as described previously [13]. Comparing the appearance of NC- and CAF-fed rats, NC-fed rats were visibly leaner than CAF-fed rats (Figure 2B) and CAF-fed rats showed increased adipose tissue deposition in the abdominal area compared to NC-fed rats (Figure 2C).

**Figure 3:**
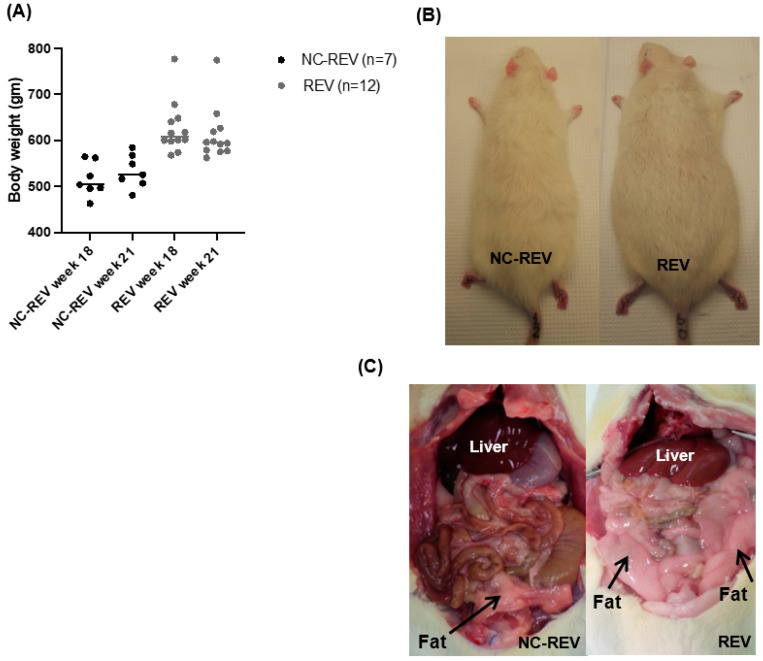
Effect of diet switch on body weight. (**A**) REV rats lost 6% of their initial weight when their diet was switched to NC. NC rats continue to gain weight as part of their physiological growth. None of the changes in either the NC or REV group were statistically significant. Data were analyzed by one-way ANOVA and expressed as the mean ± SEM. (**B**) External body size of representative animals from the NC and CAF groups; REV rat still appeared bigger compared to the NC fed-rat. (**C**) REV rats showed abdominal fat deposition, apparent in the abdominal cavity compared to the NC-fed rats. Black arrows are pointing at the fat tissues in the abdominal cavity.

The effect of CAF withdrawal for three weeks (week 21) showed a modest loss (6%) in body weight (Figure 2A), but this was not statistically significant compared to CAF-fed rats at 18 weeks, and remained higher (*p* = 0.04) than in the NC-fed rats, which displayed modest weight gain weight at 21 weeks (Figure 3A). There was no acute change in body size and abdominal adipose deposition on CAF withdrawal (Figure 3B,C).

### 3.2. Food and Water Consumption

**Figure 4:**
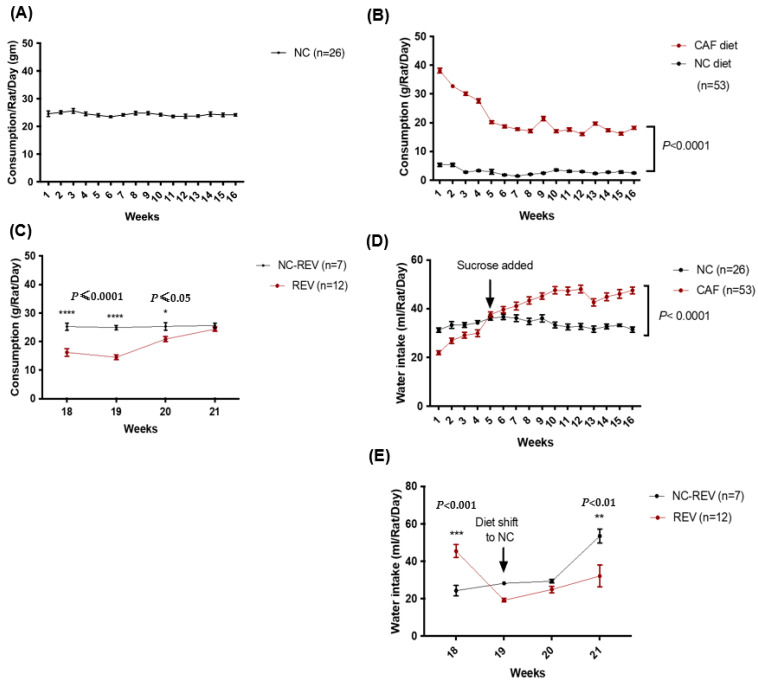
Food consumption of the NC, CAF and REV groups. (**A**) The curve illustrated constant food consumption (25 gm/rat/day) by the NC group throughout this study. (**B**) The graph represents the consumption of both diets (NC and CAF) offered for the CAF group (n = 53) in the same cage. CAF-fed rats consumed a large quantity of the CAF diet (red curve) in the beginning of this study, and consumption declined dramatically to reach approximately 20 gm/rat/day by week 5. NC consumption (black curve) for the same group was very low, approximately 5 gm/rat/day. The difference between the curves was significant *p* < 0.0001. (**C**) A significant decline in food consumption was observed in the REV rats when their diet was changed from CAF to NC, *p* < 0.0001. However, in weeks 2 (week 19) and 3 (week 20), food consumption by the REV rats increased to equal those of NC rats, approximately 25 gm/rat/day, by week 4 (week 21). (**D**) The red curve represents the water intake for CAF-fed rats, and the black curve represents the control NC-fed rats. Sucrose was added to the water of the CAF group at week 5, which dramatically increased the water intake, while NC showed steady intake. The difference between the curves was significant with a *p* value < 0.001 at weeks 8 and 9, and with a *p* value < 0.0001 starting from week 9 to week 16. (**E**) REV rats witnessed a significant drop in the water intake at week 19; this drop was reversed by week 21. NC-REV rats consumed an equal amount of water, with an unexplained rise at week 21. Data were analyzed by two-way ANOVA with repeated-measures and expressed as the mean ± SEM. * *p* ≤ 0.05, ** *p* < 0.01; *** *p* < 0.001, **** *p* ≤ 0.0001.

NC-fed animals showed constant food consumption, approximately 25 g/rat/day throughout the 16–18-week study period (Figure 4A). In the first week, CAF rats consumed very high amounts of the CAF diet, approximately 39 g/rat/day, which from the 5th week stabilized to reach 20 g/rat/day throughout the remainder of this study. The difference in consumption between the NC and CAF diets was statistically significant (*p* < 0.0001) (Figure 4B). When the CAF diet was omitted from the choice, the consumption of NC significantly dropped, but increased gradually, until food consumption of REV rats became comparable to the age-matched control rats (NC-REV) (Figure 4C).

NC-fed rats showed constant water intake throughout the 16–18-week study period (Figure 4D). Initially, CAF-fed rats consumed less water compared to NC-fed rats, although addition of sucrose to the drinking water at week 5 significantly increased intake among CAF-fed rats (*p* < 0.001) (Figure 4D). The caloric intake for each group was estimated to be 70.1 kcal/rat/day for the NC group and ranging between 87 and 98 kcal/rat/day for the CAF group (including the sucrose in water).In the first week of the reversibility period, REV rats showed a drop in water intake, which increased towards the end of the 3 weeks. Water intake was stable in the NC-REV rats throughout the study period, until week 21, when an increase in intake was observed (Figure 4E).

### 3.3. Metabolic Parameters

Systemic fasting, mean levels of glucose showed no differences between NC versus CAF (Table 1) despite a slight, non-significant increase in NC-REV before reversibility. Plasma insulin levels were significantly elevated in the CAF group compared to in the NC group (Table 1), and the shift in the diet from CAF to NC led to a slight decrease in insulin level, but was not statistically significant (Table 1). CAF-fed rats had significantly elevated serum triglyceride levels compared to NC-fed rats (Table 1). The hypertriglyceridemia was reversed when the animals were shifted to the NC diet (Table 1). The systemic levels of the liver function enzymes, AST, ALT and ALP, showed no statistically significant changes in the CAF-fed rats compared to in the NC-fed rats (Table 1).

### 3.4. Hepatic Steatosis Assessment

**Figure 5:**
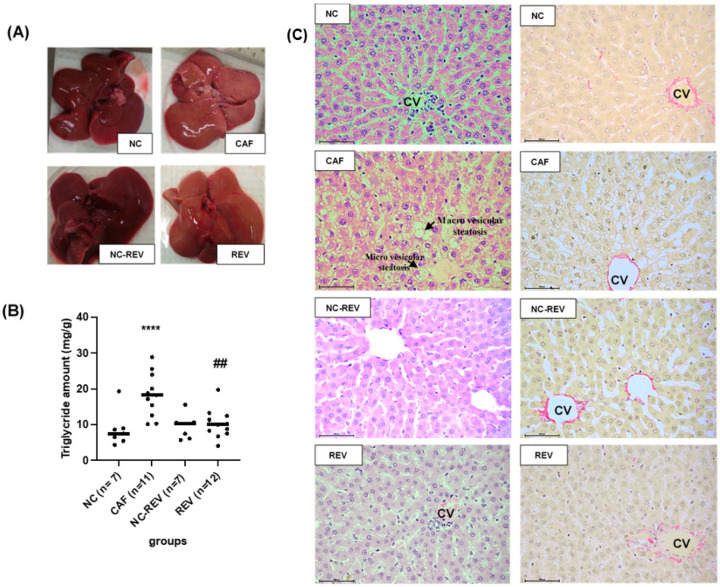
Hepatic steatosis assessment. (**A**) The liver appeared brown in color with a smooth surface in NC-fed rats, a discolored appearance was observed in CAF-fed rats, the same normal appearance was also observed in NC-REV, and discoloration was reduced in REV rat when their diet was switched to NC. (**B**) Individual plot of the triglyceride (TG) content in the liver. The CAF group showed a significant increase in TG content compared to the NC group. TG content significantly decreased with partial weight loss in the REV group when their diet switched from CAF to NC. **** represents the statistical difference (*p* ≤ 0.0001) between NC vs. CAF, ## represents the statistical difference (*p* ≤ 0.01) between CAF vs. REV. (**C**) NC, normal hepatic cells, NC (Sirius red), normal collagen fibers distribution. CAF, rats revealed hepatocyte ballooning, microvesicular steatosis and some macrovesicular steatosis around the central vein. CAF (Sirius red), no changes were observed in the collagen distribution. REV, liver showed normal hepatocytes when their diet was changed from CAF to NC. REV, no changes were observed in the collagen distribution. Images in the left panel stained with hematoxylin and eosin, and images in the right panel stained with Sirius red. Images were analyzed using 40× objective (bar = 500 px).

There were clear differences in the appearance of the livers of CAF-fed rats compared to that of the NC-fed rats. In the former group, livers were discolored compared to the denser red-colored livers from NC animals. When the CAF diet was changed to NC, the appearance was intermediate compared with the NC and CAF diets (Figure 5A). Liver weight was significantly associated with body weight. CAF-fed rat livers were significantly heavier (*p* < 0.001) compared to those of NC-fed rats. A slight reduction in liver weight was observed compared to CAF-fed liver weight, despite still being significantly higher when compared to its control NC-REV (Table 2).

Hepatic fat accumulation was evaluated by measuring tissue TG content. The TG content of the CAF-fed livers was significantly higher (*p* ≤ 0.01) compared to those of the NC-fed animals and significantly decreased (*p* ≤ 0.001) in the REV group (Figure 5B).

Histological examination showed the normal structure of hepatocytes in the control animals (Figure 5C). However, the CAF-fed group showed microvesicular fatty change in most hepatocytes, with initial macrovesicular development in some rats (Figure 5C). Changes were observed around the periportal region, the portal triad and the centrilobular region, with no signs of inflammation due to absence of inflammatory cell infiltration. A strong reversal effect was observed in the REV group when the CAF diet was changed to NC, with only grade 1 microvesicular steatosis being observed (Figure 5C) (see Supplementary Material: Table S2). Sections from the same rats were stained with Sirius red to look at the collagen distribution. Results revealed normal distribution of collagen fibers in all the studied groups (Figure 5C).

## 4. Discussion

This study described a novel DIO rodent model addressing a full period of obesity development along with a short recovery period. This study focused on the effect of carbohydrate-enriched food and drink on several precursors of metabolic syndrome (MeS), including hyperphagia, hyperinsulinemia, dyslipidemia, and fatty liver disease. The CAF food and drink were consumed with greater veracity, accompanied by increased body weight and greater accumulation of adipose tissue within omental depots and storage of triglycerides in the liver. This diet also induced hyperinsulinemia to maintain euglycemia, dyslipidemia, and hepatic steatosis. Withdrawal of the CAF diet, even over a short period, reversed most of the pathological changes, prior to significant weight loss or changes in adiposity. The CAF diet reported here appears more representative of the increased carbohydrate-rich diets consumed by people and is a robust and useful model for the study of MeS.

In the current rat model, a carbohydrate-enriched CAF diet showed significantly induced weight gain and greater accumulation of adipose tissue, both within the designated depots and ectopically in the liver. This diet produced obesity closely mimics the human condition and could therefore be used as a robust DIO model [14]. These findings are in line with previous studies that showed CAF feeding for 15 weeks induced obesity in male SD rats [15], was more effective in inducing adiposity in Wistar rats, and elevated body weight in a mouse model compared to high-fat (HF) diets [16].

Palatability is an important determinant that affects both frequency and the amount of food consumed and hence weight gain [16]. Although the CAF group was offered both CAF and NC, the consumption of the CAF diet was significantly greater than that of NC. Therefore, rodents, like human subjects, seem to prefer sweet and dense food, supporting previous results of CAF diets being more palatable than other high-fat diets [14]. The CAF diet induced hyperphagia at the start of this study, which gradually declined and stabilized until the food portion consumed by the CAF group was comparable to that consumed by the NC group, approximately 25 g/rat/day. However, the CAF diet was calorically higher. The introduction of sucrose into the water of the CAF-fed animals increased their water consumption, mimicking the intake of fizzy/soda/sugar-containing drinks by humans. The preference of the CAF diet and overconsumption of sucrose water might be related to the alteration in hormones regulating the appetite or the activation of the reward system as reported previously [17].

Adiposity is highly associated with the development of MeS. One of the key players of MeS is the disruption of the glucose–insulin axis, which is a precursor of type 2 diabetes. In this study, the CAF diet induced hyperglycemia in approximately one-third (33%) of the rats, while two-thirds (67%) of the same group exhibited hyperinsulinemia. This heterogeneity of the effects are similar to those of Sampey and colleagues who showed that 3 out of 10 CAF-fed rats were hyperglycemic after 15 weeks of the diet, suggesting a pre-diabetic state in some animals [14].

Weight gain and hyperinsulinemia are often accompanied by an atherogenic lipid profile, including elevated serum triglyceride, increased LDL-cholesterol (LDL-C), and a reduced level of HDL-cholesterol (HDL-C) [18]. In this study, the CAF diet induced a significant increase in the triglyceride levels. Physiologically, excess circulating carbohydrates are stored in the liver as either glycogen, converted to FFAs through de novo lipogenesis [19], or converted to triglycerides for longer-term storage. Among all sugar typesthat are major sources of carbohydrate, fructose is more lipogenic compared to glucose [19]. In the current study, the diet is composed of sucrose (glucose-fructose disaccharide) in both water and the CAF diet itself, suggesting that fructose has a significant contribution in inducing de novo lipogenesis. Previous reports have shown that excess carbohydrates are more readily converted to fat and stored in the form of triglycerides, mainly in adipose depots, as well as in ectopic regions, such as liver and skeletal muscles [20].

NAFLD is the most common chronic liver disease associated with MeS with hepatic steatosis being strongly associated with visceral adiposity [21]. Our results suggest that the CAF diet induced fatty liveras apparent from the appearance of the liver, with the pale color a consequence of fat accumulation in the organ. Consistent with this finding, livers of mice overexpressing human hepatic lipase were pale in color after a 16-week feeding regimen on a Western-type diet [22].Furthermore, a positive correlation between body weight and liver weight was observed which is similar to previous findings [22,23]. These results suggest that the increased liver weight may be a consequence of hepatic fat accumulation. 

Clinically, fatty liver is often diagnosed with the elevation of both ALT and AST, which agrees with studies that showed NAFLD, ALT, AST and ALP are all independent predictors for incident diabetes in humans, and further that serum ALP may be an independent biomarker of liver fibrosis in obese patients with NAFLD [24,25]. However, this study did not show changes in the levels of ALT, ALP and AST. This is consistent with NAFLD patients having a normal level of liver enzymes [26]. Furthermore, the usefulness of systemic levels of these enzymes in NASH is limited, probably due to their low specificity, sensitivity, and prognostic value [27].

The current study showed that hepatic TG was significantly elevated in CAF-fed rats compared to their control. Hepatic TG is derived from various sources, including fatty acids influx from adipose tissue (60%), de novo lipogenesis (26%) and the diet (15%) [28]. Of these, adipose tissue lipolysis and de novo lipogenesis are considered the main lipid sources in hepatic steatosis [28]. Diets rich in carbohydrates stimulate the synthesis of FFAs which are esterified to TG and form a lipid droplet in hepatocytes [20]. The CAF diet used in the current model appears to contribute significantly to hepatic TG formation.

A majority of the liver samples showed development of microvesicular lesions, with macrovesicular steatosis in some samples. These rats were diagnosed with grade 3 liver microvesicular steatosis; however, few animals developed macrovesicular steatosis around the periportal region, the portal triad and the centrilobular region. The increasing severity of NAFLD is usually associated with lobular inflammation [1]. However, in this study, CAF-fed animals, despite showing early stages of NAFLD, had no signs of inflammation. Different diets induce NAFLD to variable degrees based on the type and duration of feeding. For example, Wistar rats fed a high-fat diet for sixteen weeks showed clear liver damage [29], while high fructose levels in drinking water induced simple steatosis within 8 weeks [30]. Also, prolonged feeding with high-fat diets in mice induce the progression of NAFLD from simple steatosis to more sever stages (NASH) [31]. Thus, diet is an important mediator in the development of NAFLD.

Weight loss is an effective strategy to combat obesity-related pathologies. In the current model of a carbohydrate-enriched diet combined with a sedentary lifestyle, reducing the carbohydrate content, after induction of obesity, for a relatively short period of 3 weeks resulted in a modest, but non-significant weight loss (of 6%), caused by a dramatic drop in food and water consumption, perhaps due to low palatability of the NC diet. This weight loss was also not associated with any significant changes in the deposition of abdominal adipose tissue. Previous work suggests that a longer intervention period (1.5 months) may be needed to significantly reverse the gain in weight and adipose tissue brought about by approximately 2 months of CAF feeding [32]. However, the withdrawal of the carbohydrate component from the diet, even for such a short a period, was adequate to bring about an improvement in the hyperinsulinemia and hypertriglyceridemia. In addition, this improvement was achieved by dietary intervention alone, with the animals being relatively sedentary. Exercise has been shown to alleviate insulin resistance after CAF feeding in rats [33].

Also, the appearance of the liver improved with the reduction in serum TG. Consistent with our findings, CAF withdrawal completely reversed systemic TG in young rats [34]. Moreover, hepatic TG elevation was diminished with reversal of the liver steatosis from grade 3 to grade 1 (almost normal healthy liver). Although the CAF diet used in this study induced obesity associated with fatty liver (NAFLD), it did not reach a severe enough stage to be associated with inflammation and fibrosis. Histological assessment showed that the reversal of NAFLD and NASH depends on the severity of the disease, and as the severity increases, the chances of reversing the liver lesion is decreased [35]. This supports a previous study that showed weight reduction markedly improved steatosis [36], suggesting that weight loss is one of the best treatment for obesity-induced NAFLD and NASH [11].

Hepatic fat accumulation is regulated through different mechanisms such as de novo lipogenesis and fatty acid uptake or decreased lipid export through fatty acid oxidation. The results presented here showed that feeding with a high-carbohydrate diet induced hepatic fat accumulation, which was reversed with dietary intervention. However, the mechanistic pathways that mediates these changes require more investigation.

## 5. Conclusions

In conclusion, a DIO rodent model fed with a carbohydrate-enriched diet led to many features of MeS, including hyperinsulinemia, hypertriglyceridemia, and hepatic steatosis. Dietary intervention by a reduction in this macronutrient was able to restore normoinsulinemia, and reverse some of the obesity-related hepatic abnormalities, even in the absence of significant weight loss. Further investigation is needed to clarify the metabolic pathways involved in the progression and reversal of abnormalities, particularly with regard to liver function and NAFLD.

## Figures and Tables

**Table 1:** Effect of the cafeteria (CAF) diet and diet switch on metabolic parameters levels.

(A)				
Parameter	Obese		Reversible	
	NC (n = 11)	CAF (n = 12)	NC-REV (n = 6)	REV (n = 10)
Cholesterol (mmol/L)	1.77 ± 0.125	1.77 ± 0.107	1.88 ± 0.13	2.1 ± 0.15
HDL (mmol/L)	1.44 ± 0.107	1.11 ± 0.08	1.58 ± 0.13	1.66 ± 0.12
LDL (mmol/L)	0.29 ± 0.04	0.26 ± 0.04	0.35 ± 0.058	0.35 ± 0.044
Triglycerides (mmol/L)	0.904 ± 0.127	1.75 ± 0.19 ***	0.82± 0.2	1.1 ± 0.12 ^##^
ALP (U/L)	68.91 ± 4.57	89.9 ± 5.44	71.89 ± 7.5	62.03 ± 3.9
ALT (U/L)	79.66 ± 9.77	83.48 ± 28.77	123.3 ± 16.80	83.22 ± 9.24
AST (U/L)	362.77 ± 56.21	295.61 ± 52.65	372.7 ± 70.32	292.95 ± 35.24
Glucose (mmol/L)	9.79 ± 0.35	9.8 ± 1.1	12.9 ± 0.8	10.4 ± 0.9
**(B)**				
**Parameter**	**Obese**		**Reversible**	
	**NC** **(n = 7)**	**CAF** **(n = 7)**	**NC-REV** **(n = 4)**	**REV** **(n = 6)**
Insulin (ng/mL)	0.58 ± 0.08	1.19 ± 0.2 *	0.79 ± 0.13	1.0 ± 0.15

(**A**) Lipid profile, liver function enzymes and glucose for obese and reversible rats. CAF-fed rats had significantly elevated serum triglyceride levels compared to NC-fed rats, and this reversed when the animals were shifted to the NC diet. (**B**) Plasma insulin level was significantly increased when rats were fed with the CAF diet for 16–18 weeks compared to NC; this was slightly, but not significantly, decreased in the REV rats compared to in the NC-REV rats. Data were analyzed by one-way ANOVA and expressed as the mean ± SEM. * represents the statistical difference between NC vs. CAF, # represents the statistical difference between CAF vs. REV. * *p* ≤ 0.05, ## *p* ≤ 0.01, *** *p* ≤ 0.001. Abbreviations: HDL, high-density lipoprotein; LDL, low-density lipoprotein; ALP, alkaline phosphatase; ALT, alanine aminotransferase; AST; aspartate transferase.

**Table 2:** Liver weight and body weight (wt) for obese and reversible rats.

	CAF		REV	
Parameter	NC (n = 11)	CAF (n = 15)	NC-REV (n = 7)	REV (n = 12)
Body wt (g)	555.4 ± 14.34	646.56 ± 16.54 ***	532.85 ± 13.66	612.54 ± 16.54 *
Liver (g)	12.66 ± 0.42	17.36 ± 0.87 ****	12.73 ± 0.8	15.62 ± 0.74 *

Data were analyzed by one-way ANOVA and expressed as the mean ± SEM. * represents the statistical difference between NC vs. CAF, or NC-REV vs. REV. * *p* ≤ 0.05, *** *p* ≤ 0.001. **** *p* ≤ 0.0001.

## Data Availability

All relevant data are contained within this article. The original contributions presented in this study are included in this article/supplementary material; further inquiries can be directed to the corresponding author.

## Supplementary Materials

The following supporting information can be downloaded at: https://www.mdpi.com/article/10.3390/nu16010115/s1, S1: CAF diet preparation; Table S1: Nutritional contents for food items; Table S2: NAFLD scoring results for the NC, CAF and REV groups.
