# Supplementary material for: Relative Recovery of Non-Alcoholic Fatty Liver Disease (NAFLD) in Diet-Induced Obese Rats
Source: Nutrients. 2023 Dec 28;16(1):115. doi: 10.3390/nu16010115 (PMC10780646; doi:10.3390/nu16010115)
Supplement: Supplementary file 1 [file nutrients-16-00115-s001.zip › nutrients-2692263-supplementary.pdf]

Supplementary Material

Relative recovery of Non-alcoholic fatty liver disease (NAFLD) in diet-induced obese rats

Hamda M. Aboujassoum*, Vidya Mohamed-Ali, David Abraham, Lucie H. Clapp, Hamda A. Al-Naemi

*** Correspondence:** Hamda M. Aboujassoum: hbojassom@qu.edu.qa

S1: Supplementary Data: CAF Diet Preparation

CAF diet characterized by high carbohydrates was used in this study. The food items used in the study were available in the local market and consumed by human subjects. The selection was initially based on the food label, and then these food items were sent to the food analysis laboratory (Central Food Laboratories, Public Health Department, Ministry of Health -Qatar) to confirm the nutritional contents. The final selection of the food items was based on the lab analysis results except the Macaroni, because it was added in the middle of the study. Despite the other nutritional contents of the diet, in this study we focused only on the percentage of macronutrients: Carbohydrate, fat and protein.

Diet was prepared freshly every day using the following food items: frozen burger, sliced bread, macaroni, mini croissant (Table 1). CAF diet was prepared as mixture of two items from Table 1, and served as a homogenized meal. The carbohydrate content was completed by adding 5% sucrose in drinking water. All mixtures were provided equally (every day) in a raw form without cooking in a sterilized plate inside the cage. Rats were offered the three food mixtures in different durations. In the beginning of the study the food was changed in week 9, then food was changed between the 3 combinations every 2 weeks throughout the study to sustain increased food consumption.

Table S1: Nutritional contents for food items.

Table represents the nutritional content for each food item used in the study from lab analysis and the commercial label.

Parameter	Beef burger 1		Beef burger 2		Mini Croissant		Milk slice bread		Macaroni	
	2 kcal/g		2.2 kcal/g		3.8 kcal/g		2.6 kcal/g		3.6 kcal/g	
	Lab analysis (%)	Commercial label (%)	Lab analysis (%)	Commercial label (%)	Lab analysis (%)	Commercial label (%)	Lab analysis (%)	Commercial label (%)	Lab analysis (%)	Commercial label (%)
**Moisture content**	24	-	19	-	24.6		34.5		NA	-
**Ash content**	5.8	-	4	-	1.20		1.2		NA	-
**Fat content**	20	16.16	6	9.7	15	15	1.4	1.8	NA	1.7
**Fiber content**	2.2	-	4	-	3.8	4.5	3	2.9	NA	2
**Protein**	12		14.4	15.6	8	10	6.3	8.9	NA	12
**Carbohydrate**	36.0	40	52.6		47.4	52	53.6	56.4	NA	71

Table S2: NAFLD scoring Results for NC, CAF and REV groups.

group	Animal #	Score				
		Hepatocyte Ballooning	Microviscular steatosis	Macroviscular steatosis	PMNC	MNC
NC	1	0	1	0	0	0
	2	0	0	0	0	0
	3	0	1	0	0	0
	4	0	2	0	0	0
CAF	1	1	1	0	0	0
	2	1	3	1	0	0
	3	1	3	2	0	0
	4	1	1	0	0	0
	5	1	3	1	0	0
	6	0	2	0	0	0
REV	1	0	0	0	0	0
	2	0	1	0	1	1
	3	0	1	0	0	0
	4	0	2	0	0	0
	5	0	0	1	0	0
	6	0	1	0	0	0

Notes: Ballooning (hypertrophy) of hepatocytes, was scored based on the number of hypertrophic hepatocytes per field: 0 (absent), 1 (minimal), 2 (moderate) and 3 (sever). Steatosis was divided into two categories: microvesicular and macrovesicular and was evaluated on the percentage of the affected areas: 0 (5%), 1 (5–33%), 2 (34–66%) and 3 (≥.66%). Inflammation was determined based on the number of infiltrated inflammatory cells per foci (focal liver lesion): 0 (absent), 1 (minimal), 2 and 3 (moderate) 4 (sever).
